# Reproducible propagation technique for the symbiotic cnidarian model system *Cassiopea xamachana*

**DOI:** 10.1242/bio.059413

**Published:** 2022-09-13

**Authors:** Casandra Newkirk, Sankalp Vadlapudi, Mahita Sadula, Cheri Arbello, Tingting Xiang

**Affiliations:** Department of Biological Sciences, The University of North Carolina at Charlotte, Charlotte, NC 28223, USA

**Keywords:** Cassiopea, Cnidarian-algal, Jellyfish, Symbiosis

## Abstract

The phylum Cnidaria is composed of corals, jellyfish, hydras, and sea anemones. Cnidarians are well-known for their regenerative capability, with many species maintaining the ability to regenerate complete structures. This regenerative capacity has been used casually for propagation purposes (via dissection) for some cnidarians used in laboratory research but has yet been documented in a manner meant to be reproducible. One such cnidarian model system is the scyphozoan jellyfish *Cassiopea xamachana*. *C. xamachana* has become an emerging model system for studying the cnidarian-algal symbiotic relationship, so determining a reliable and fast method for expansion of laboratory animals is crucial. Here we outline a reproducible propagation method for continued generation and growth of *C. xamachana* polyps.

This article has an associated First Person interview with the first author of the paper.

## Abstract (precis)

**Summary:** This manuscript outlines a dissection protocol to propagate the upside-down jellyfish, *Cassiopea xamachana*.

## INTRODUCTION

Many species in the phylum Cnidaria (e.g. corals, sea anemones, and jellyfish) maintain a symbiotic relationship with dinoflagellate algae in the family Symbiodiniaceae (Davy et al., 2012; LaJeunesse et al., 2018). Studies involving this symbiosis have employed many different model systems to study questions regarding the establishment and maintenance of algae in host cells along with the breakdown of the relationship under stress (a phenomenon known as bleaching) (Dunn et al., 2002; Davy et al., 2012; Bucher et al., 2016). One such model system that has gained traction in the last few years is the scyphozoan jellyfish *Cassiopea*. *Cassiopea* has a typical scyphozoan life cycle including: a larval stage, a polyp stage, an ephyra stage, and an adult medusa stage (Hofmann et al., 2003). *Cassiopea* has many attractive features as a model system to study cnidarian symbiosis including: (1) polyps can be rendered completely free of symbionts, (2) polyps can be imaged neatly on microscope slides, (3) animals can be reared and maintained in a laboratory setting, (4) *Cassiopea* is not an endangered species like many symbiotic coral species, and (5) most *Cassiopea* species are readily available in the field (Newkirk et al., 2018; Newkirk et al., 2020; Jinkerson et al., 2022; Muffett et al., 2022).

Due to the emerging prominence of *Cassiopea* as a model system, a method to obtain a large quantity of animals (in particular juvenile polyps) to perform experiments is necessary. While there are current ways to increase the number of *Cassiopea* in a laboratory setting, they can be slow in some cases and require very controlled conditions. One of the main methods of producing a greater number of *Cassiopea* for experimental use is via the asexual budding of the polyps. High levels of budding, settlement of the buds, and the metamorphosis of the buds into juvenile polyps typically requires conditions such as bacterial inducers from natural seawater and a stable temperature range (<20°C) (Fitt et al., 1987; Fitt and Costley, 1998). These precise conditions are not always present in a laboratory setting, especially in situations where artificial seawater that has been autoclaved and filtered is used, removing any potential natural settlement inducers (personal observation). It can also take a good deal of time to produce polyps that are large enough to perform certain experiments, such as those requiring inoculation with algae (personal observation). This can be especially true if there is a lack of suitable substrate for the buds to settle and attach to (Hofmann et al., 1978). In another popular system, the sea anemone *Exaiptasia*, one method of propagation is the cutting of polyps to generate two equal halves that will regenerate to create two whole and separate animals (Singer, 1971). Because of the similarities of *Cassiopea* in its polyp stage to *Exaiptasia* we sought to test the same cutting methods utilized, and to establish a new propagation method for *Cassiopea*.

Cnidarians as a whole have extensive regenerative capabilities, with even the smallest fragments of some species having the capacity to regrow into a full animal (with some species being able to regenerate from muscle isolates and dissociated cells) (Galliot and Schmid, 2003; Leclère and Röttinger, 2017). This quality has been used in many different species to propagate animals for laboratory experiments (Bode, 2003; Bradshaw et al., 2015; van der Burg and Prentis, 2021). Scyphozoans as a class have different levels of regenerative properties. *Aurelia aurita*, the moon jellyfish, has been one of the better studied scyphozoans in terms of regeneration. Studies have found that *Aurelia* polyps can regenerate into whole animals from tiny fragments, and adult *Aurelia aurita* exhibit a self-repair mechanism known as symmetrization when body parts are amputated (Steinberg, 1963; Abrams et al., 2015). Other scyphozoan species, such as *Mastigias* and *Chrysaora*, have displayed symmetrization following cuts during the ephyra stage (Fujita et al., 2021). Studies involving *Cassiopea* species have found that these jellyfish are capable of regeneration at each life cycle stage. Planula can regenerate into whole polyps from small fragments, polyps can regenerate new structures from the oral end, and medusa can regenerate missing structures (albeit not as well as during earlier life stages) (Curtis and Cowden, 1972; Neumann, 1979; Gamero-Mora et al., 2019). Though the regenerative capability of *Cassiopea* has been outlined in the literature, no published work has shown if the cutting propagation method used for cnidarian model systems such as *Exaiptasia* would work similarly as a reliable and rapid means of producing more *Cassiopea* polyps. In this paper we provide a proper method to cutting *Cassiopea* aposymbiotic polyps that will allow the animals to regenerate properly and be used for future experiments. This algal free polyp type is of most interest to us as several studies are performed in which aposymbiotic polyps are inoculated with different Symbiodiniaceae strains to observe phenotypes or responses to environmental stimuli. We also outline preliminary results on how cutting impacts *Cassiopea* polyps that are infected with algae and how cutting impacts *Cassiopea* at the juvenile ephyra stage.

## RESULTS AND DISCUSSION

### Regeneration of tentacles and oral arms

**Fig. 1.**
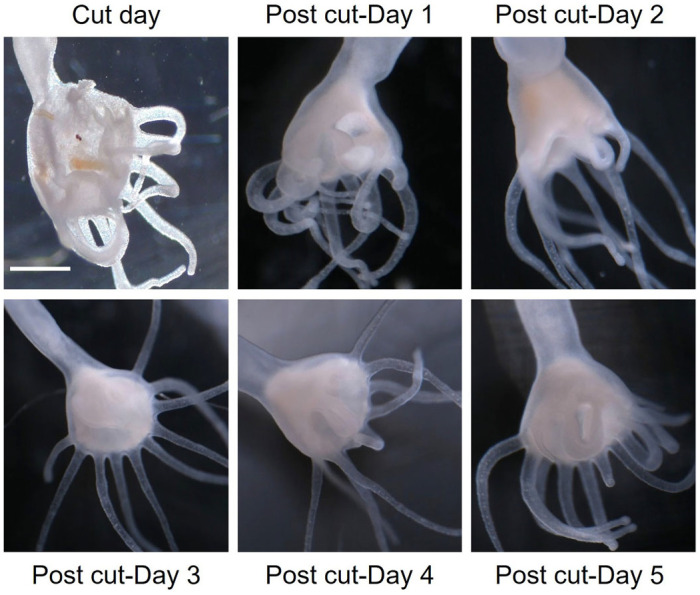
**Wound healing in aposymbiotic polyps.** Wound healing for cut site 5 days post-cut. Scale bar: 500 µm.

**Fig. 2.**
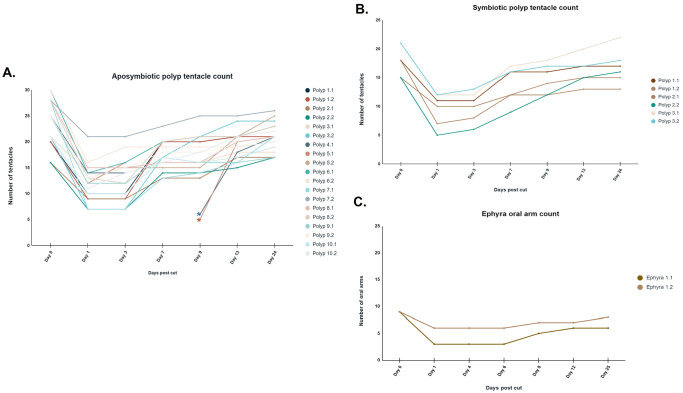
**Polyp tentacle count and ephyra oral arm count following cut.** (A) Count of tentacle growth of the two halves of three cut aposymbiotic polyps over a 24-day period. (B) Count of tentacle growth of the two halves of one cut symbiotic polyp over a 24-day period. (C) Count of oral arm growth of the two halves of one cut ephyra over a 25-day period. *Polyps that were stressed on day 7 and tentacle count was not obtained; count continued on day 9 following recovery.

**Fig. 3.**
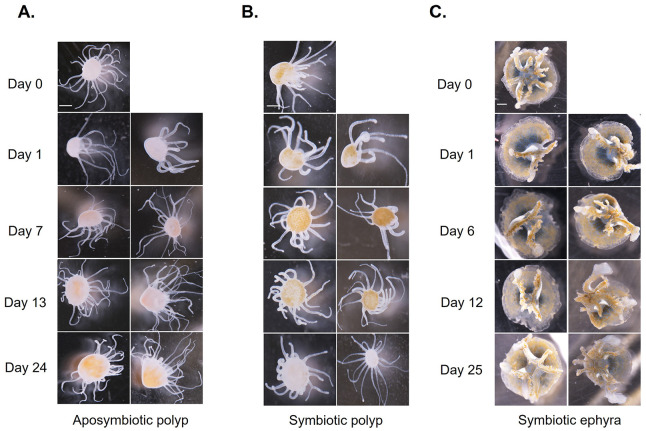
**Tentacle regeneration of aposymbiotic and symbiotic polyps over a 24-day period.** (A) Images of growth of tentacles in a cut aposymbiotic polyp over a 24-day period. (B) Images of growth of tentacles in a cut symbiotic polyp over a 24-day period. Scale bars: 500 µM.

**Fig. 4.**
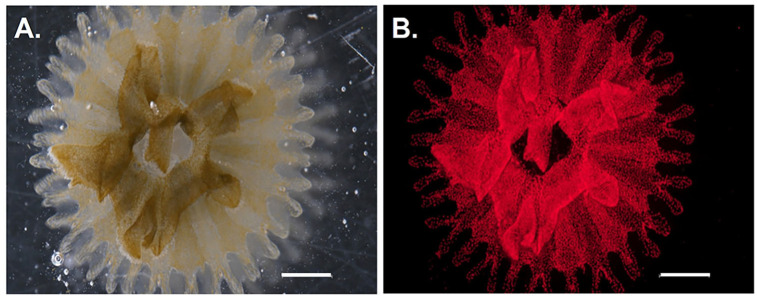
**Strobilation of symbiotic polyp.** Ephyra strobilated from recovered SSB01 infected cut polyp. Scale bars: 1000 µm. (A) Brightfield capture of SSB01 ephyra. (B) Autofluorescence of algal chlorophyll in SSB01 ephyra.

In the first 3 days following the cut in both the ten aposymbiotic polyps and the three symbiotic polyps, the tentacle numbers for each polyp half remained the same or close to the same as directly post-cut. Also 3 days post cut in the aposymbiotic polyps the wound site was completely closed from the incision, and the animals continued to regenerate tentacles (Fig. 1). Tentacle numbers then increased to around the same as pre-cut between days 7 and 9 in aposymbiotic polyps (Fig. 2). In aposymbiotic polyp halves 24 days following the cut, each half had regenerated most of the lost tentacles, with the halves of a few of the polyps exceeding the number of tentacles from the starting count (Figs 2A and 3A). Aposymbiotic polyp 4 became stressed during the experimental timeframe due to a bacterial contamination, which lead to the death of half 4.2 by day 7 and half 4.1 not able to be counted on day 7 but ultimately recovering over the rest of the 24 days. Aposymbiotic polyp 5.1 was also stressed due to the same circumstances and was not counted on day 7, but ultimately recovered. These two stressed aposymbiotic polyp halves were still able to regenerate a tentacle count close to the tentacle number before dissection (Fig. 2A). In symbiotic polyp halves, tentacle counts were close to or the same as starting counts by day 7 (Figs 2B and 3B). Cut symbiotic polyps were also able to continue through the life cycle and strobilate, as seen by the produced ephyra in Fig. 4. Polyps had the capacity to eat *Artemia* provided to them one day post cut. In contrast, the two ephyra halves regenerated fairly slowly over the 25-day time frame, and by day 25 the oral arm count of the two halves were close to the same number as the starting count but did not exceed it (Figs 2C and 3C). The ephyra halves were fed *Artemia* one day post cut. This difference between the polyp stage and the ephyra stage to regenerate quickly and efficiently is likely due to the decrease in regenerative capability in more advanced stages of the life cycles (Curtis and Cowden, 1972; Gamero-Mora et al., 2019).

### Statistical analysis

A paired *t*-test was conducted on each group (aposymbiotic polyps, symbiotic polyps, and ephyra). This was done to compare the difference of the number of tentacles and oral arms before and after cutting and regeneration. For aposymbiotic polyps the *P*-value was 0.0117, for symbiotic polyps the *P*-value was 0.1801, and for ephyra the *P*-value was 0.2952. The *P*-values here show that there is a statistically significant difference between original tentacle count and the tentacle count after regeneration for aposymbiotic polyps, but no statistically significant difference for tentacle or oral arm number before and after cutting for symbiotic polyps and ephyra. This analysis provides overall data that polyps and ephyra after regeneration had tentacles counts similar to that of polyps after cutting.

### Relevance to laboratory work

The regeneration of *Cassiopea* polyp tentacles and structures following a cut, approximately 7 days, will be a valuable laboratory tool moving forward. After 7 days, the newly halved aposymbiotic polyps can be used for experimental purposes and have shown the same aptitude for taking in algae as in uncut polyps. The growth of aposymbiotic polyps can be seen in the increase of our aposymbiotic colony that was expanded over the course of 40 days (Table 1). The polyp count presented in Table 1 displays polyp numbers that do not include the ten aposymbiotic polyps presented in our regeneration data but show the increase of our aposymbiotic polyp colony using the dissection method described here. Though there are other ways to propagate *Cassiopea* polyps (asexual budding and settlement of sexual planula), these can require very specific cues (either bacterial or temperature wise) in a laboratory setting. Buds and planula meant for settling are at times subject to death and degradation under non-optimal conditions such as temperatures outside of the 25-27°C range and seawater that does not contain as the right bacterial settlement cues (such as autoclaved seawater) (Neumann, 1979; Ohdera et al., 2018). Cutting allows for a fast and reliable way to generate high polyp numbers and could also be useful in creating clonal lines for algal infection experiments. Polyps that are cut maintain the ability to asexually bud, so continued growth of buds via that method would still be possible if preferred for certain experiments. Though regeneration of ephyra is much slower than seen in either aposymbiotic or symbiotic polyps, it does occur and can be useful in the long term for generating *Cassiopea* at more advanced life cycle stages. Overall, this paper presents another method that can be used to maintain large quantities of aposymbiotic *Cassiopea* and preliminary data on how this method can be utilized on other *Cassiopea* life stages and of different algal infection status. This method will allow us another avenue for growing this emerging model symbiotic cnidarian in the laboratory for use in experimental studies.

**Table 1.**
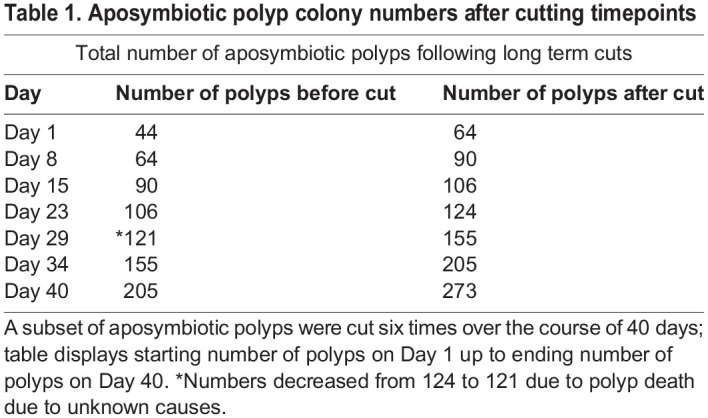
Aposymbiotic polyp colony numbers after cutting timepoints

## MATERIALS AND METHODS

### Preparation for cut and imaging

*Cassiopea* polyps were kept in six-well plates in 30 ppt artificial seawater (ASW) for maintenance throughout the experiment. Aposymbiotic polyps were maintained in complete darkness at 27°C, while symbiotic polyps and ephyra were maintained in programmed chambers at 50 μmol photons m^−2^ s^−1^ of PAR light levels at 27°C for the duration of the experiment. Polyps and ephyra were fed twice weekly with *Artemia*, with water changes occurring 24 h after feeding. Imaging for both aposymbiotic and symbiotic polyps was done on microscope slides. Coverslips placed over polyps were raised using modeling clay so the animals would not be flattened, and animals were arranged in a small drop of ASW so that all tentacles could be seen and counted. Ephyra were immobilized in ASW in 24-well plates using a few drops of a 1:1 ASW mixed with 0.37 M MgCl_2_ solution. The ephyra was imaged directly in the 24-well plate. Animals were imaged using a Nikon SMZ25 microscope. Day 0 images displayed the whole animal before the cut and were necessary to obtain the initial number of tentacles or oral arms.

### Cutting and post-cut maintenance

Polyps and ephyra were transferred to the lid of a Petri dish following imaging, and any excess seawater was removed from the Petri dish when necessary to help with the precision of the cut. A razor blade was employed to make a vertical incision down the middle of the polyps and through the mouth area of the ephyra splitting the bell and oral arms into two halves. The razor blade was pressed firmly during the process to ensure a clean and complete cut. When the incision was complete, the two new halves of the animal were placed back into separate wells of a six-well plate (making sure to differentiate between each half). Aposymbiotic polyp halves were placed in complete darkness at 27°C and held under these conditions for the remainder of the counting period. Symbiotic polyp halves and ephyra halves were placed at 27°C under 12:12 light conditions for the remainder of the counting period. Both polyps and ephyra halves were fed *Artemia* two times weekly. For this experiment, ten aposymbiotic polyps were cut while three symbiotic polyps infected with SSB01 algae were cut (larger polyps with ≥15 tentacles were used to ensure a more precise cut). SSB01 algae were originally isolated from an *Exaiptasia* clonal line (Xiang et al., 2013), and infected into *Cassiopea* polyps to establish a colony of animals inoculated with that strain in our laboratory. In addition to the aposymbiotic and symbiotic polyps cut, one 6-month-old ephyra known to be hosting its native symbiont *Symbiodinium microadriaticum* (Jinkerson et al., 2022) was also cut to observe oral arm regeneration. The two halves of the ten cut aposymbiotic polyps and three cut symbiotic polyps were imaged over the course of 24 days to obtain the tentacle counts of each half. Images were taken on Day 0, Day 1, Day 3, Day 7, Day 9, Day 13, and Day 24. The two ephyra halves were imaged over the course of 25 days with images being taken on Day 0, Day 1, Day 4, Day 6, Day 8, Day 12, and Day 25. Images were used to ensure the animal halves recovered well and regenerated completely.
